# An Increase of the Character Function of Self-Directedness Is Centrally Involved in Symptom Reduction during Remission from Major Depression

**DOI:** 10.1155/2011/749640

**Published:** 2011-11-24

**Authors:** Jaap G. Goekoop, Remco F. P. De Winter, Rutger Goekoop

**Affiliations:** ^1^Department of Psychiatry, Leiden University Medical Centre, 2300 RC Leiden, The Netherlands; ^2^Psycho-Medical Centre, Parnassia, Monsterseweg 93, 2553 RJ The Hague, The Netherlands; ^3^Programma Depressie-Ambulant, Parnassia, PsyQ, Lijnbaan 4, 2512 VA The Hague, The Netherlands

## Abstract

*Background*. Studies with the Temperament and Character Inventory (TCI) in depressive disorders have shown changes (Δ) of the character of Self-Directedness (SD) and the temperament of Harm Avoidance (HA). The central question of this study is which of these two changes is most proximally related to the production of depressive symptoms. *Methods*. The start and endpoint data from a two-year followup of 58 depressed patients were reanalyzed. We used the ΔHA and ΔSD scores as well as the Δ scores on three dimensions of psychopathology, called Emotional Dysregulation (ED), Retardation (RET), and Anxiety (ANX). The presence of the main relation between personality and psychopathology was tested in all patients and in four subcategories. The data were analyzed by MANCOVA and Structural Equation Modelling (SEM). *Results*. ΔHA and ΔSD correlated negatively, and only ΔSD was related (negatively) to ΔED. This pattern was found in all subcategories. SEM showed ΔHA and ΔSD had an ambiguous causal interrelationship, while ΔSD, ΔRET, and ΔANX had unidirectional effects on ΔED. *Conclusion*. The results correspond with a central pathogenetic role for a state-related deficit at the character level in depression. This may have important consequences for investigations of endophenotypes and clinical treatment.

## 1. Introduction 

A change of personality has been found consistently in major depressive episodes [1]. A central question is whether this should be seen as an epiphenomenon or an essential step in the pathogenetic process. The current study focuses on changes of personality and relations with changes in the production of depressive symptoms in the course of remission. In order to allow for a fine-grained analysis of the personality changes involved, and for an optimal detection of relations with dimension(s) of psychopathology, we used multidimensional rating scales. The choice of dimensions for personality and psychopathology to be considered is important in such analyses. This will be discussed here below.

Previous studies of personality in patients with a major depressive disorder have shown that the premorbid personality traits of Neuroticism [2], Harm Avoidance (HA) [1, 3], and Self-Directedness (SD) [3] are related to the life-time risk of a depressive episode. Since Neuroticism is positively correlated with HA and negatively with SD [4], whereas HA and SD are themselves negatively correlated [5–17], these findings suggest that HA and SD represent different aspects of the more global vulnerability or resilience trait, that is nonspecifically covered by the Neuroticism dimensions of several other personality models [18–20]. Since Neuroticism does not predict the time of onset of the depressive episode [21], this global dimension may not be sufficiently differentiated to allow for the detection of the most proximal personality dimension that, in interaction with stress, would be involved in the eventual pathogenesis of the depressive disorder. For this reason, we used the Temperament and Character Inventory (TCI) [5] with its differentiation between SD and HA in this global domain of personality. In order to enhance the chance of finding the dimension that is most proximally related to the transition from normal to pathological functioning and therefore to the production of depressive symptoms, we used state-related changes. 

Changes of personality have been found before to be related to changes of depression in varying degrees of severity, and the findings may vary depending on the use of the measures of personality change. The first to mention are relations between subsyndromal symptom production and changes of Neuroticism immediately above the basal level [22]. In the higher severity range of symptom production changes of Neuroticism have been found to be present [23] but small [24], while highly reproducible changes have been found for the HA and SD dimensions of the TCI [1, 25–28]. The varying frequency of “comorbid” Axis-II diagnoses in patients with major depression [29] could be a third way in which personality changes may be assessed. From the perspective of the TCI, low basic levels of SD are the defining hallmark of personality disorders [30]. As improvement of the level of depressive symptoms has been found to correlate with the change in Axis-II prediction based on this SD score [25], the state-related-reduced SD in depression may be involved in this Axis-II “comorbidity”. These findings support the necessity to differentiate between the dimensions of SD and HA in the studies of the primary and most proximally related factor in the onset and remission of depressive disorders. In the present study we therefore used the change of both dimensions, hypothesizing that either ΔHA or ΔSD would be most directly involved in the production of symptoms in depressive disorders. The analyses were carried out in all depressed patients as well as in four subcategories to test if the relation between the change of personality and change of psychopathology found is a general characteristic of all depressive disorders or just pertains to one or more subcategories.

The phenotypical significance of ΔHA or ΔSD can be derived from the personality model of the TCI and the subscales that are comprised by these dimensions. According to the TCI [5], personality can be conceived as a multidimensional construct comprising higher and lower levels of personal functioning and coping called character and temperament respectively. Whereas character is thought to involve conscious-adaptive information processing, temperament involves automatic adaptation via conditioned response patterns. The model includes three character dimensions called Self-Directedness (SD), Cooperativeness (CO), and Self-Transcendence (ST) and four temperament dimensions called Harm Avoidance (HA), Reward Dependence (RD), Novelty Seeking (NS), and Persistence (PER). HA comprises the subscales or facets of worrying/pessimism, fear of uncertainty, shyness, and fatigability, while low SD results in apathy, a loss of goals or direction, loss of self-striving behaviour, externalizing, and an incongruent second nature. This suggests that either or both changes could be directly involved in the pathogenesis of depression or one or more subcategories in particular.

To optimize the chances of finding relations with specific aspects of major depression, we also used a multidimensional approach to assess psychopathology. This involved the administration of the Comprehensive Psychopathological Rating Scale (CPRS) [31], which enables the assessment of six global dimensions of psychopathology [32] called Emotional Dysregulation (ED), Motivational Inhibition (or retardation (RET), Autonomous Dysregulation (or anxiety (ANX), Motivational Disinhibition (or Mania), Perceptual Disintegration, and Behavioural Disintegration. For the present study we used the three nonpsychotic and non-manic global dimensions of ED, RET, and ANX. Emotional Dysregulation (ED) is a 20-item scale that comprises 9 of the 10 items of the Montgomery Asberg Depression Rating Scale (MADRS) [33]. Other items of the dimension of ED concern specific neurotic symptoms like compulsive thoughts, phobias, indecision, fatigability, failing memory, reduced sexual interest, reported muscular tension, loss of sensation or movements, derealisation, and depersonalisation [32]. The dimension of RET comprises items of inability to feel, apparent sadness, observed lack of appropriate emotion, reduced speech, and slowness of movement. The dimension of ANX comprises items of inner tension, reduced sleep, reported autonomic disturbances, aches and pains, observed autonomic disturbances signs, and observed muscular tension [32]. We used these global dimensions of psychopathology in the present study as we previously have found combinations of ANX and RET to be specifically involved in the phenotypes of subcategories of depression derived from the melancholic subtype [34]. This method has also enabled the detection of a phenotypic homology between one of these subcategories called depression with above-normal vasopressin concentration [35] and the stress-induced behavioural pattern of the animal model for depression called high anxiety-like behaviour rat [36]. Moreover, the combination of ED and RET appeared to be involved in psychotic depression [37].

As has already been reported previously [1], we investigated the changes of personality and psychopathology in the context of a two-year follow-up study of patients treated for an acute episode of major depression. We used the change scores between the start and the end of this two-year follow-up period. We first analyzed the correlations between the changes of the dimensions of personality and the dimensions of psychopathology by using Pearson's correlations and MANCOVA. Thereafter, we used Structural Equation Modelling (SEM) to analyze the pathway between personality change and change of psychopathology and at the same time the pathways between the changes of the dimensions of psychopathology. Since the personality dimensions of character and temperament and the dimensions of psychopathology represent different levels of functioning from the conscious conceptual level of character via the temperamental level of automatic conditioned behaviour to instinctual response patterns, the results of the present study are discussed from the perspective of the hierarchic organization of brain regions involved in depression. The support for either of two pathogenetic models will be evaluated. These models are based on the hypothesis of a continuity between premorbid temperament, increased temperament score, subsyndromal symptom level, major depressive disorder [38, 39], and the hypothesis of the development of a high-level functional deficit as precondition for the production of depressive symptoms [40–42]. Since support has been found for high HA as the most general premorbid temperament and for low SD as additional vulnerability factor for just a subcategory of depression [1], we consider the continuity model to be supported if ΔHA relates most directly with the change of psychopathology, and the high-level functional deficit model to apply if ΔSD is most directly related to the change of psychopathology.

## 2. Methods

### 2.1. Subjects

We used the data set from 58 depressed patients who completed a two-year followup [1]. Mean age was 39.1 (*sd*⁡ = 11.8) years, 40 (69.0%) were female, 35 (60.3%) were outpatients, and 49 (84.5%) were at least partially remitted. Forty-one patients (70.7%) had depression in full remission, 8 patients (13.8%) had depression in partial remission, and 9 (15.5%) still fulfilled criteria for major depressive episode. The level of education was assessed from low education = 1 to level 6 = university or postgraduate. The mean level of education was 3.3 (*sd*⁡ = 1.6). 

The group of 58 patients was divided into four subcategories. These subcategories were based on our previous studies of vasopressinergic and noradrenergic mechanisms in depression and subcategories in the field of melancholic or endogenous depression [34] and psychotic depression [37]. These studies have resulted in two subcategories, called Highly Anxious-Retarded (HAR) depression, depression with above-normal plasma AVP concentration (ANA), as well as in support for psychotic depression as a distinct subcategory. In the present study, we eliminated all overlap between these three subcategories. This resulted in the following four subcategories: (1) psychotic depression (according to the DSM-IV-TR) (*n* = 7), (2) nonpsychotic depression with above-normal plasma AVP concentration (ANA-R) (*n* = 12), (3) nonpsychotic normal AVP highly anxious-retarded depression (HAR-R) (*n* = 12), and (4) all other depressed patients (*n* = 27).

### 2.2. Assessments

#### 2.2.1. Personality

As in our previous studies on depression [1, 43], we used the Dutch translation [6] of the Temperament and Character Inventory (TCI) [5]. The lists were filled in within 2 weeks after recruitment and every 6 months until 2 years after recruitment. Patients were asked to respond to the items “as if they were in their premorbid state”, to maximally reduce state-dependent changes of response tendency.

#### 2.2.2. Psychopathology

We used three of the six global dimensions of psychopathology assessed by the Comprehensive Psychopathological Rating Scale (CPRS) [31, 32]. These were the basic nonpsychotic and nonmanic dimensions of Emotional Dysregulation (ED), Motivational Inhibition (or psychomotor retardation (RET)), and Autonomic Dysregulation (or somatic anxiety (ANX)). We excluded the manic and two psychotic dimensions because these symptoms dimensions were not supposed to contribute to a large degree to the differentiation between the clinical pictures. 

### 2.3. Statistical Analyses

Pearson's correlations were used to test the correlation between ΔSD or, ΔHA and, ΔED, ΔRET, or ΔANX. Bonferroni correction was used, and alpha was set at *P* < 0.0083 to correct for 6 assessments. Two MANCOVAs were used to analyse the dependence of ΔED, ΔRET, and ΔANX on ΔSD and ΔHA. In these analyses, we changed the positions of the two sets of variables as dependent and independent variables. Sex was used as independent factor, and age and levels of education as covariates in an additional analysis. These analyses were carried out with the SPSS version 18.0. 

A combined method was used with partial correlations (PC) and Structural Equation Modelling (SEM) to construct a graph and to analyze the correlation coefficients between the nodes and the weights of the edges, to explore the causal direction of the dependencies found between the changes of all dimensions of personality and psychopathology. This analysis was carried out using TETRAD, a software package for causal analyses provided by Carnegie Mellon University [44].

## 3. Results

### 3.1. Means and Δ Scores of HA, SD, ED, RET, and ANX

Table 1 shows means and standard deviations of the scores at start and after 2 years of followup on the personality dimensions of SD and HA and the basic symptom dimensions of ED RET, and ANX. The two personality dimensions and the three dimensions of psychopathology changed significantly over the two years. SD increased while all other dimensions decreased. 

### 3.2. Correlations between ΔSD, ΔHA, ΔED, ΔRET, and ΔANX

Table 2 shows the correlations between the changes of all dimensions of personality and psychopathology used in this study. In all 58 patients, there was a moderately high negative correlation between ΔSD and ΔHA, a moderate negative correlation between ΔSD and ΔED, and a low positive correlation between ΔHA and ΔANX (just lacking statistical significance after Bonferroni correction), while there were moderately high positive correlations between ΔED, ΔRET, and ΔANX. The difference in the strength of the correlations of the latter three scores of the change of psychopathology suggests that the ED functions as the central or common dimension of psychopathological change. 

### 3.3. Dependence of ΔED, ΔRET, and ΔANX on ΔSD and ΔHA, and Vice Versa

MANCOVA (Table 3) with ΔED, ΔRET, and ΔANX as dependent variables and ΔSD and ΔHA as independent variables showed that the relation between change of character (ΔSD) or temperament (ΔHA) and change of psychopathology was restricted to the relation between ΔSD and ΔED. The addition of sex, age, and level of education did not result in a significant relation with any of the two dimensions of personality change. MANCOVA with ΔSD and ΔHA as dependent variables and ΔED, ΔRET, and ΔANX as independent variables (Table 4) shows that the strength of the relation between ΔSD and ΔED increased if this relation was controlled for the effect of ΔRET and ΔANX on ΔED.

**Figure 1:**
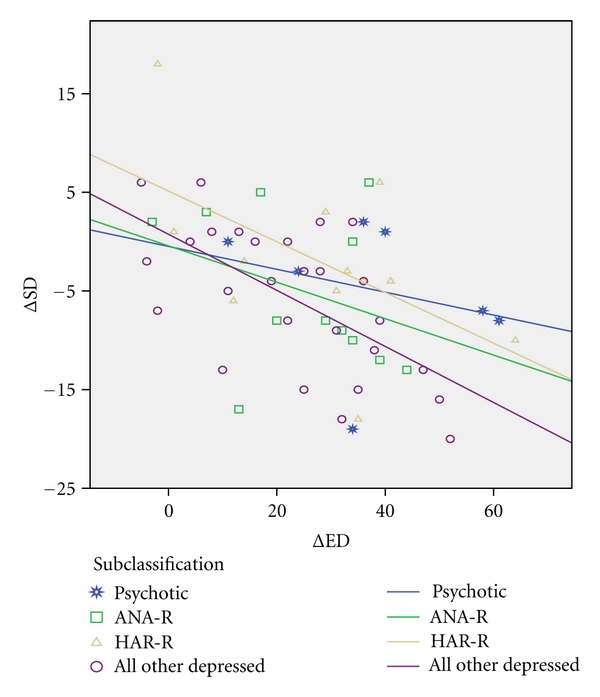
Negative relations (lines corresponding with regression coefficients) between the change of Self-Directedness (ΔSD) and the change of Emotional Dysregulation (ΔED) in 4 subcategories of depression. (1 = psychotic depression, 2 = ANA-R, 3 = HAR-R, and 4 = all other depressed patients).

If the differentiation into 4 subcategories was added as fixed factor to this MANCOVA model, then it appeared that the strongest correlation was found in the largest subcategory of all other depressed patients (*F* = 9.303; *P* = 0.004) and that not any of the three other subcategories had a significantly deviant correlation. The subcategory of HAR-R depression, which has the lowest SD score after full remission had a nearly significantly higher range for ΔSD (*t* = 1.877; *P* = 0.066) than the group of All Other Depressed patients with one patient having a high decrease of SD after 2 years (see Figure 1). 

### 3.4. Pathways Involved in Symptom Production

**Figure 2:**
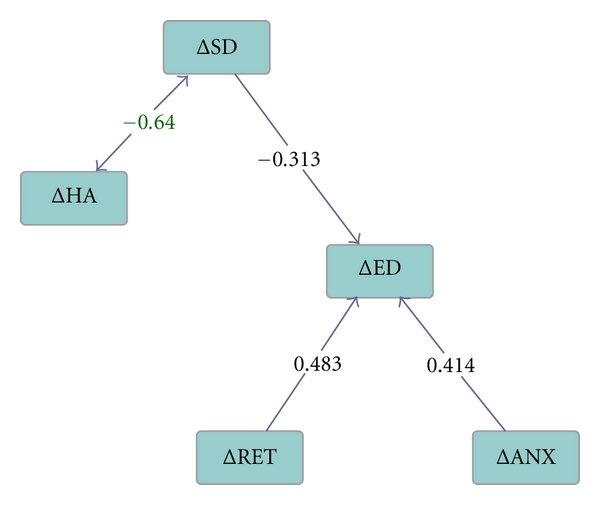
Pathways assessed by Structural Equation Modelling of relations between change scores over two years for the personality dimensions of Harm-Avoidance (ΔHA) and Self-Directedness (ΔSD), and the dimensions of psychopathology of Emotional Dysregulation (ΔED), Retardation (ΔRET) and Anxiety (ΔANX) in 58 patients with major depression. The numbers represent correlation coefficients, except for the ambiguous relation between ΔSD and ΔHA, which is expressed in terms of the covariance coefficient.

Structural Equation Modelling (Figure 2) showed that ΔHA and ΔSD were bidirectionally related, suggesting the possibility of a positive feedback loop within the change of personality associated with the production of depressive symptoms. As far as the relation between the two domains of personality change and depressive symptoms is concerned, ΔSD was related uniquely and negatively with ΔED (*P* = 0.0024), and this involved a causal effect of ΔSD on ΔED, but not vice versa. ΔRET and ΔANX were uninfluenced by ΔHA and ΔSD, and each had a unique positive contribution to ΔED (*P* = 0.001 and 0.010 resp.). Figure 2 shows the correlations. The Edge Coefficients of the weights of the effects were as follows: ΔHA on ΔSD –0.32 (SE = 0.13), ΔSD on ΔHA –0.38 (SE = 0.09), ΔSD on ΔED −0.58 (SE = 0.18), and ΔRET and ΔANX on ΔED 1.89 (SE = 0.45) and 1.23 (SE = 0.35), respectively. 

## 4. Discussion

### 4.1. Correlations

As previously reported on data from the same patient sample [1] the present study showed that the mean of the score of the character dimension of SD increased during the two-year followup, while the mean of the score of the temperament dimension of HA showed a decrease. The present study now in addition showed that the state-related changes of SD and HA were negatively correlated, and that only ΔSD correlated with the change of psychopathology. This ΔSD appeared to correlate uniquely with the change of the psychopathology dimension of ED. Within the domain of psychopathology the changes of all three dimensions (ED, RET, and ANX) appeared to be strongly intercorrelated, with ΔED having the strongest correlations. This suggests that this dimension of ED represents the core of the depressive disorder and that ANX and RET are variably associated dimensions, as has been found in our previous studies on the clinical phenotype of subcategories of depression [35, 37, 45]. 

### 4.2. SEM Findings and Support for the High-Level Functional Deficit Model

The bidirectional pathway between ΔSD and ΔHA, combined with the absence of a correlation between ΔHA and any dimension of psychopathology, corresponds with a relatively independent dynamic interaction within the field of personality. As the dimension of HA is thought to represent a conditioned sensitivity for stressful events and SD a learned way to cope with stress conditions [5], this bidirectional relation could reflect a stress-induced vicious circle of the experience of stress, a loss of learned coping, and an increase of the sensitivity for stress conditions. The unidirectional causal pathway between ΔSD and ΔED suggests that the stress-induced loss of SD may function as a central pathogenetic factor for the production and maintenance of depressive symptoms. Since the state relatedness of reduced SD has been found in all of the 4 subcategories in which we divided the whole group of depressive disorders, and ΔSD and ΔED now appeared to be correlated in all these subcategories, this factor appears to be a general characteristic of major depression. The hierarchic structure found by SEM of the relations within the psychopathological domain between ΔED, ΔRET, and ΔANX may correspond with a recent model of activated regions within the hierarchic organization of brain structures involved in the “default resting state” of depression [39]. 

The results of the present study do not support the model of a continuity between premorbid temperament, increased temperament, subsyndromal symptom level, and major depressive disorder. In contrast, the relation of ΔSD to ΔED corresponds with the classic high-level functional deficit model of mental disorders [41], derived from neurology [40]. While this high level deficit has more recently been claimed to apply specifically to neuropsychological functional deficits of depressive disorders [42], we now found evidence that it may be conceptualized in terms of psychological functioning. According to the classic model, a high-level functional deficit (described as “negative” symptoms) should be the actual pathogenetic factor that functions as the precondition for relatively lower level functions to become disinhibited and to produce the most manifest or “positive” symptoms of the disorder. ΔSD can be conceived as such a high-level functional deficit, and ΔED, ΔRET, and ΔANX as dimensions of psychopathology that result from the disinhibition of lower levels of cerebral organization. 

This ΔSD may be a useful target for the translational search for endophenotypes of depressive disorders. This means that the accidentally discovered inability of the HAB rat—an animal model with increased vasopressinergic activation and increased vulnerability for depression—to activate the Dorso-Medial Prefrontal region that is normally involved in the inhibition of conditioned avoidance behaviour, may be seen as such an endophenotype [46]. In contrast, the hypotheses that the depressive disorder can be conceived as a severe form of the premorbid trait or temperament [38, 47], or as being due to an abnormally increased activation of a network that is also activated by a normal affective response to stress in healthy brains [39], would direct the search for endophenotypes towards regions of the brain that could not be most centrally involved in the pathogenetic mechanism of depression. 

Since the diagnosis of an Axis-II disorder depends on low SD [30], and the change of SD during the change of depression has been found to reduce the prediction of an Axis-II diagnosis in a substantial way [25] around the mean of the frequency of the Axis-II diagnosis in depression [27], the present support for a central role of ΔSD in the pathogenesis may result in a revision of the interpretation of this Axis-II diagnosis from a secondary complication or “comorbidity” [29] to a change of personality that is inherently related to the general and central pathogenetic mechanism. This reinterpretation of the clinically obvious and disturbing deficit of Axis-II “comorbidity” will also probably enhance the interest in related neurobiological changes and targets for treatment. 

The unidirectional effects from ΔRET and ΔANX on ΔED may be due to several factors. These effects could be inherently related to a sequential pattern of remission in clinical pictures of subcategories of depression with high ANX and/or RET, like HAR depression and psychotic depression [37, 45]. On the other hand, dimensions of RET and ANX could be influenced independently by specific treatments. 

### 4.3. Implications for the Neurobiological Research of SD

The central role for ΔSD in the relation between change of personality and psychopathology, and the negative interaction between ΔSD and ΔHA suggest that the hypofunctional and hyperactive cerebral regions involved in this relation should be investigated in detail. Up to now only evidence has been reported of a relation between the character dimension of SD and “prefrontal function” [48, 49], while HA has been found to be related with more specifically defined regions, like the right Anterior Cingulum [50] and the Subgenual Anterior Cingulate Cortex (SUACC) [51]. Three regions, the Perigenual Anterior Cingulate Cortex (PACC), the (SUACC), and the Ventro-Medial Prefrontal Cortex (VMPFC), have consistently been found to be abnormally activated during a depressive state [39]. This suggests that one should search for neurobiological correlates of ΔSD in a network that complementarily mediates at the highest prefrontal level both the balance between activation and inhibition of conditioned emotional responses and the top-down regulation of the lower-level neurobiological correlates of emotional, instinctual and neuroendocrine states. This network could comprise the already-mentioned Dorso-Medial Prefrontal Cortex (DMPFC), which inhibits emotional and conditioned responses and has been found to be hypofunctional in depressed patients [52]. The same network could also comprise the medial prefrontal/cingulate region that is involved in the inhibition of the glucocorticoid response to stress [53], and the ventromedial prefrontal region [54] that is involved in the extinction memory of conditioned freezing behaviour. A problem with the supposedly reduced function of the DMPFC in depression is that a stress-induced increased activation of this region has been found in depression and that this was found to be associated with HA [55]. This suggests that future studies should carefully delineate in what extent the responses and state-related activities of the DMPFC are related to both SD and HA. 

A limitation of the present study is that it only supports the central role of reduced SD in the pathogenesis of depression in the second part of the acute episode during the transition from full pathology to remission. The findings therefore warrant investigations of the first part of the acute episode of depression. Nonetheless the support for a central role of reduced SD in the pathogenesis of depression warrants further research-related prefrontal hypofunction and treatment effects both in man and translational studies of animal models of depression. 

## Figures and Tables

**Table 1:** Mean scores (standard deviation between brackets) of SD, HA, ED, RET, and ANX at the start and after two years, and their differences, in 58 patients with depression.

	Number	Mean score at start	Mean score at end of 2-year followup	Difference between start and end of followup	*P*-value of the difference
SD	58	23.7 (7.0)	28.4 (7.9)	4.74 (7.70)	<0.001
HA	58	25.4 (5.8)	23.2 (7.4)	−2.16 (6.44)	0.014
ED	58	52.8 (11.6)	26.6 (16.4)	−26.21 (16.74)	<0.001
RET	58	8.5 (3.4)	3.8 (3.8)	−4.74 (3.64)	<0.001
ANX	58	11.3 (4.0)	7.4 (4.5)	−3.90 (4.80)	<0.001

**Table 2:** Correlations between the changes of (Δ) the dimensions of personality (Self-Directedness and Harm Avoidance) and the dimensions of psychopathology (Emotional Dysregulation, Retardation, and Anxiety) in all 58 patients (lower left part of the table).

	Δ Self-Directedness	Δ Harm Avoidance	Δ Emotional Dysregulation	Δ Retardation	Δ Anxiety
Δ Self-Directedness					
Δ Harm-Avoidance	**−,641**				
	<.001				
Δ Emotional Dysregulation	−,**463**	,330			
	<.001	,011			
Δ Retardation	−,211	,173	**,677**		
	,111	,195	<.001		
Δ Anxiety	-,313	,339	,**679**	,**594**	
	,017	,009	<.001	<.001	

**Table 3:** The dependence of the change of (Δ) Emotional Dysregulation, Δ Retardation and Δ Anxiety on Δ Self-Directedness, and Δ Harm Avoidance in 58 patients assessed over 2 years (*F* and *P* values of a MANCOVA).

	Δ Emotional Dysregulation	Δ Retardation	Δ Anxiety
Δ Self-Directedness	7.54 (0.008)	0.994 (0.323)	0.990 (0.324)
Δ Harm Avoidance	1.35 (0.715)	0.135 (0.714)	2.048 (0.158)

**Table 4:** The dependence of the change of (Δ) Self-Directedness and Δ Harm Avoidance on Δ Emotional Dysregulation, Δ Retardation, and Δ Anxiety in 58 patients assessed over 2 years (*F* and *P* values of a MANCOVA).

	Δ Emotional Dysregulation	Δ Retardation	Δ Anxiety
Δ Self-Directedness	9.58 (0.003)	1.43 (0.237)	0.078 (0.781)
Δ Harm Avoidance	1.88 (0.176)	0.783 (0.380)	2.014 (0.162)
